# The complete chloroplast genome of a Korean endemic species *Sophora koreensis*, Nakai

**DOI:** 10.1080/23802359.2020.1797579

**Published:** 2020-07-29

**Authors:** Rahul Vasudeo Ramekar, Eun Ju Cheong, Hwa Lee, Kyong-Cheul Park, Myounghai Kwak, Ik-Young Choi

**Affiliations:** a Department of Agriculture and Life Industry, Kangwon National University, Chuncheon, South Korea; b Division of Forest Science, Kangwon National University, Chuncheon, South Korea; c Plant Resources Division, National Institute of Biological Resources, Incheon, South Korea

**Keywords:** Chloroplast genome, *Sophora koreensis*

## Abstract

In this study, we report the complete chloroplast (cp) genome of *Sophora koreensis* and its relation with other species within the Fabaceae family. The cp genome was 154,870 bp long, with a typical quadripartite structure including a pair of inverted repeat regions (25,866 bp) separated by a large (85,037 bp) and small (18,101 bp) single-copy (SC) region. The genome encodes a total of 84 protein-coding genes, 35 tRNA genes, and 8 rRNA genes. Phylogenetic analysis suggested that *S. koreensis* is closely related to genus *Sophora alopecuroides var. alopecuroides* within Fabaceae.

*Sophora koreensis*, commonly known as ‘Geanusam’, is an endemic species in Korea that belongs to the Fabaceae family; it is a deciduous shrub mainly found in the mountainous area of the Korean Peninsula (Lee et al. 2004). Although ‘Geanusam’, was classified separately as *Echinosophora koreensis*, it was debatable and recently merged with genus *Sophora* (Jang et al. 2019). Both roots and flowers of *S. koreensis* are rich resources of alkaloids (Murakoshi et al. 1985), flavonoids (Choi et al. 2009), and isoflavonoids (Iinuma et al. 1991) adding medicinal value against microbes and fungus (Sohn et al. 2004). Studies suggest that *S. koreensis* is effective in preventing hangover after alcohol intake (Choi et al. 2009). Although *S. koreensis* was delisted as protected and endangered wild species, the number of natural population has depleted rapidly due to overexploitation and environmental changes. The extant population is fragmented and under increased threat, yet we know little about its genetic background.

The chloroplast (cp) genome is present at a high copy number and is much smaller than the nuclear genome. The conserved gene structure and sequence divergence between species make cp genome ideal candidates for phylogenetic, taxonomic, and genetic studies (Ravi et al. 2008). Here, we report on the complete cp genome sequence of *S. koreensis* to provide an essential genetic resource and reveal the phylogenetic relationships of this species within the Fabaceae family. Plant material was collected from its natural habitat in Yanggoo, Gangwon-do, South Korea (voucher number: NIBRVP0000729369), and total genomic DNA was extracted from fresh leaf tissue using a DNeasy Plant mini kit (Qiagen, Hilden, Germany). The genome was sequenced using the HiSeq 4000 platform. The raw reads were quality trimmed using Trimmomatic (Bolger et al. 2014) and assembled using SPAdes (v3.11.1). We used the DOGMA program (Wyman et al. 2004) and ARTEMIS software (Rutherford et al. 2000) for performing annotation, and tRNAscan v1.21 (Schattner et al. 2005) to verify all tRNA genes. We have submitted the assembled and annotated sequence to GenBank under accession number MT571487.

**Figure 1.**
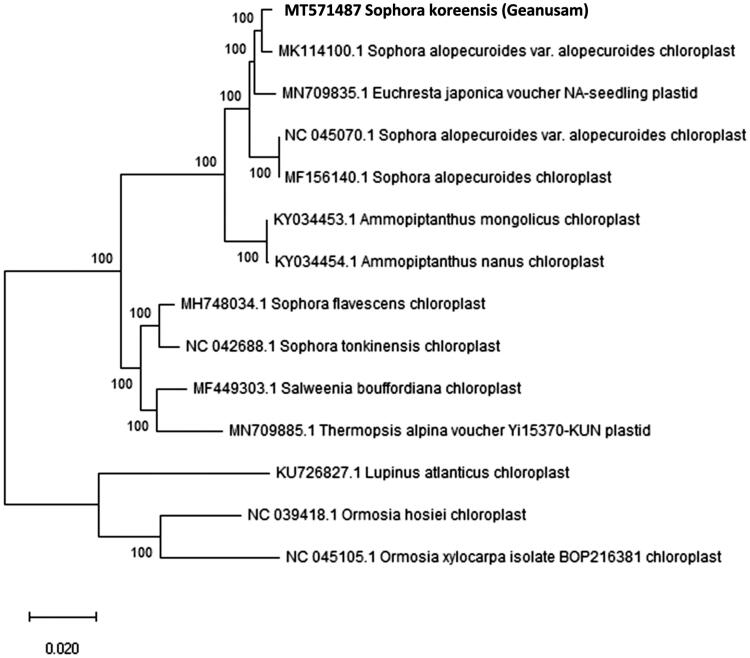
Molecular phylogenetic tree of the Fabaceae family based on the complete cp genome of 14 species.

To investigate the phylogenetic status of *S. koreensis*, the complete cp genome of 13 species within the Fabaceae family was selected. We constructed a neighbor-joining (NJ) tree with Mega 6.0 using 1000 bootstrap replicates (Tamura et al. 2013) (Figure 1). It clustered the species into two major groups, all the members of tribe Sophora (*S. koreensis*, *Sophora alopecuroides var. alopecuroides*, *Euchresta japonica*, *Sophora alopecuroides*, *Ammopiptanthus mongolicus*, *Ammopiptanthus nanus*, *Sophora flavescens*, *Sophora tonkinensis*, *Salweenia bouffordiana*, and *Thermopsis alpina*) clustered in one group. Another group with species *Lupinus atlanticus*, *Ormosia hosiei*, and *Ormosia xylocarpa* were placed in a distinct cluster. *S. koreensis*, along with *Sophora alopecuroides var. alopecuroides*, formed a monophyletic clade with a high bootstrap value, indicating a close relationship among these species.

## Data Availability

The data that support the findings of this study are openly available in GenBank of NCBI at https://www.ncbi.nlm.nih.gov, reference number MT571487.
